# Prognostic factors associated with a stable MR4.5 achievement in chronic myeloid leukemia patients treated with imatinib

**DOI:** 10.18632/oncotarget.23691

**Published:** 2017-12-26

**Authors:** Massimo Breccia, Matteo Molica, Gioia Colafigli, Fulvio Massaro, Luisa Quattrocchi, Roberto Latagliata, Marco Mancini, Daniela Diverio, Anna Guarini, Giuliana Alimena, Robin Foà

**Affiliations:** ^1^ Hematology, Department of Cellular Biotechnologies and Hematology, Sapienza University, Rome, Italy

**Keywords:** chronic myeloid leukemia, deep molecular response, imatinib, prognosis, discontinuation

## Abstract

Deep molecular response in chronic myeloid leukemia (CML) patients treated with imatinib is a prerequisite for possible discontinuation. We identify clinico-biologic features linked with the probability of reaching MR4.5 (BCR-ABL/ABL ≤ 0.0032% IS) as a stable response (confirmed on two or more consecutive determinations). In a series of 208 patients treated with imatinib first-line outside clinical trials, after a median follow-up of 7 years the incidence of stable MR4.5 was 34.6%, obtained in median time of 5.4 years. In univariate analysis, female gender (*p* = 0.02), lower median age (56.4 vs 58.6, *p* = 0.03), Sokal risk stratification (*p* = 0.01) and e14a2 type of transcript (43% vs 31%, *p* = 0.02) are associated to achievement of a stable MR4.5. In multivariate regression analysis, female gender (HR 1.6, 95% CI: 1.1–2.6; *P =* 0.022), Sokal risk (HR 1.4, 95% CI: 1.1–2.3; *p* = 0.03), type of transcript (e14a2 vs e13a2 type, HR 1.6, 95% CI: 1.3–2.9; *P =* 0.03) and achievement of an early molecular response (EMR) at 3 months (HR 1.5, 95% CI: 1.2–2.8; *P =* 0.01), retained statistical significance. These clinical and biologic features associated with the achievement of a stable deep molecular response should be taken into account at a time when treatment-free remission strategies are being actively pursued in the management of CML.

## INTRODUCTION

The introduction of tyrosine kinase inhibitors (TKIs) into the pharmacologic armamentarium for the treatment of chronic myeloid leukemia (CML) has drastically changed the management and outcome of chronic phase patients [1]. With imatinib, the first TKI approved, an improved overall survival (OS) has been reported in patients who obtain a complete cytogenetic response (CCyR) within 12 months from the start of treatment [2]. In addition, a significantly reduced rate of progression occurs in patients who achieve the “safe haven” of a 3-log molecular reduction, namely a major molecular response (MMR) (BCR-ABL/ABL ratio < 0.1%), within 18 months [2]. Achievement of deep molecular responses opens the way to attempt a possible treatment discontinuation over time. Different clinical trials have shown that about 40% of patients treated with imatinib may remain in molecular remission after discontinuation, without transformation into blast crisis or acquisition of resistance [3, 4]. Second-generation TKIs, dasatinib and nilotinib, as frontline treatment can increase the rates of patients who achieve a deep molecular response and thus also increase the possibility of discontinuing treatment over time [5,6].

More robust and standardized definitions of molecular response have been introduced and are universally accepted [7]. Patients who reach a transcript level of ≤ 0.01% obtain a 4-log reduction (MR4), whereas a BCR-ABL1/ABL ratio of ≤ 0.0032% identifies a 4.5-log reduction (MR4.5). Both identify a deep molecular response and the latter degree of response is defined as a complete molecular response. Patients who achieve a deep molecular response have a better outcome, with an improved event-free survival (EFS), progression-free survival (PFS) and OS, and a low risk of progression and disease relapse [8–10]. Factors affecting achievement of a deep molecular response have been reported in a few studies, mostly based on patients enrolled in clinical trials [8, 9]. Aim of our study was to analyze the rate of stable MR4.5 in a large series of CML patients in chronic phase treated first-line with imatinib in a real life setting at a single center and outside of clinical trials. Factors that predict the likelihood of achieving a stable MR4.5 response have been also investigated.

## RESULTS

### Characteristics of population

**Figure 1:**
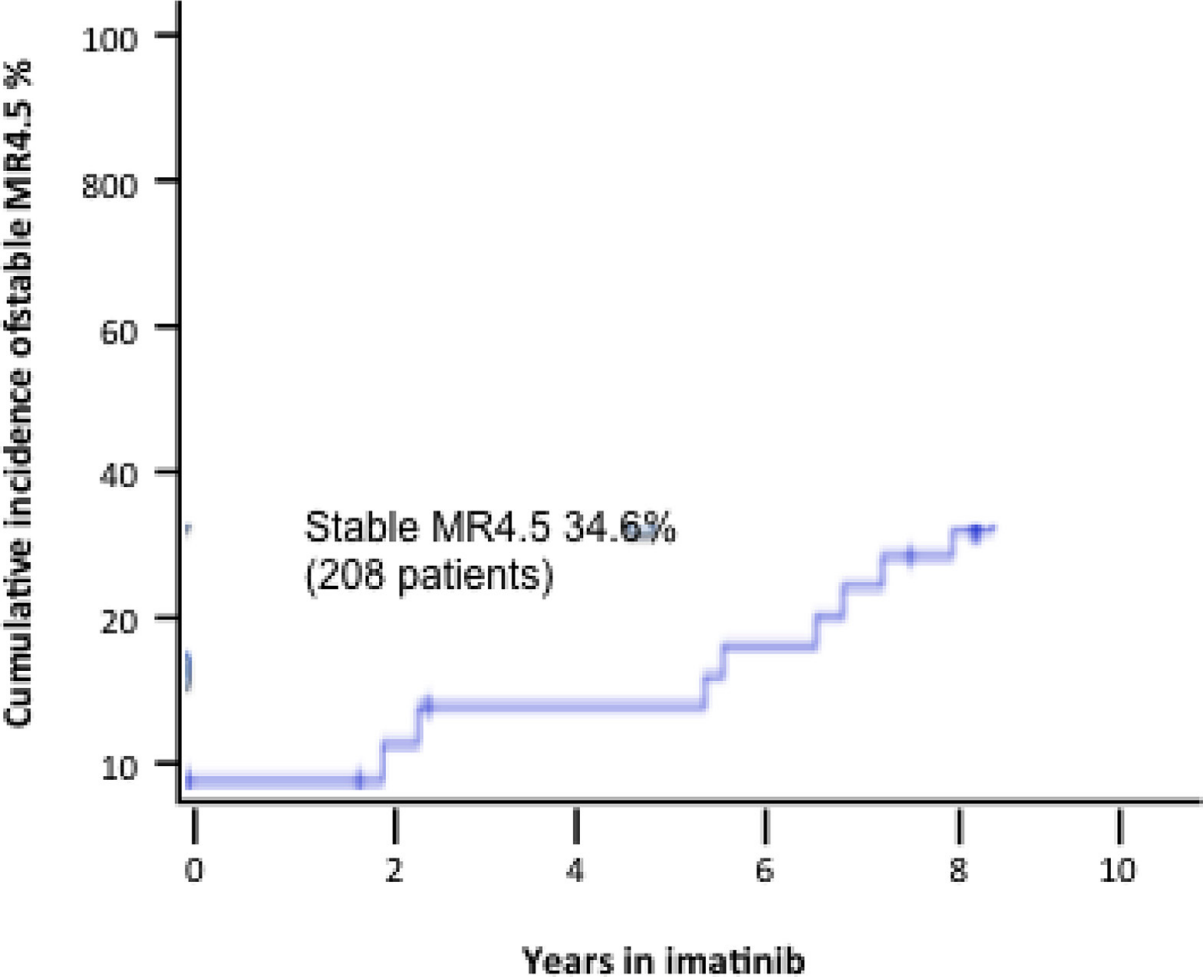
Cumulative incidence of stable MR4.5 in 208 patients treated with imatinib frontline

Overall, we retrospectively analyzed 208 patients; median age was 57.7 years (range 21–90) with 65% of patients > 65 years old. Sokal risk stratification stratified 100 patients as low risk (48.1%), 83 as intermediate (39.9%) and 25 as high risk (12%). According to the European treatment and outcome study (EUTOS) stratification, 114 patients were low risk (54.8%) and 94 high-risk (45.2%). Overall, after 1 year the incidence of CCyR was 83%, whereas at 7 years the cumulative incidence of MR3 was 70% and of MR4 51.5%. The overall incidence of a stable MR4.5 was 34.6% (Figure 1), obtained after a median of 5.4 years from the start of treatment. The incidence of primary cytogenetic resistance was 12% and of secondary resistance 13%; 3% of patients progressed to blast phase. In the entire population, the median OS at 7 years was 81.5% and the EFS 65% (Table 1).

**Table 1:** Clinical features and responses of the case series analysed (208 patients)

Features	No. (%)
Age (median)	57.7
Sokal risk Low Intermediate High	100 (48.1%) 83 (39.9%) 25 (12%)
Type of transcript e13a2 e14a2 Both	97 (46.6%) 108 (51.9%) 3 (1.4%)
Eutos risk Low High	114 (54.8%) 94 (45.2%)
Cumulative incidence of CCyR (at 1 year)	83%
Cumulative incidence of MR3 (at 7 years)	70%
Cumulative incidence of MR4 (at 7 years)	51.5%
Cumulative incidence of MR4.5 (at 7 years)	48%
Primary resistance	12%
Secondary resistance	13%
BC progression	3%
OS	81.5%
EFS	65%

### Univariate analysis results

We analyzed in univariate analysis the prognostic variables associated with the achievement of a stable MR4.5 (Table 2): we identified a significant difference in the rate of MR4.5 reached according to gender (female 48% compared to 31% in male patients, *p* = 0.02) and median age (56.4 in patients who achieved a stable MR4.5 vs 58.6 in patients who did not, *p* = 0.03). Moreover, we found a significant association between the Sokal risk stratification and the likelihood of obtaining a MR4.5: 32% in patients classified as low risk, 22% in intermediate risk patients and 11% in high risk patients (*p* = 0.01). The e14a2 type of transcript was associated with a significantly increased rate of MR4.5 (43%) compared to the e13a2 type (31%, *p* = 0.02). At 3 months, in the cohort who reached a stable MR4.5 only 1% of patients had not achieved an early molecular response (EMR or BCR-ABL1/ABL ratio < 10%), compared to 6% in the cohort that did not achieve a stable MR4.5 (*p* = 0.001). The rate of MR3 at 3 months was 15% in the cohort of patients who achieved stable molecular response vs 4% in the cohort who did not (*p* = 0.02). At 6 months, a MR3 was obtained by 43% of the patients who achieved a stable MR4.5 compared to only 19% in the cohort who did not reach this stable response (*p* = 0.01). At 12 months, this difference was again significant (69% vs 26%, *p* = 0.001). None of the patients who achieved a stable MR4.5 developed resistance with an EFS of 100%, whereas the EFS in the cohort who never achieved a stable MR4.5 was 81% (*p* = 0.01). Indeed, as previously reported, fluctuations of the molecular residual disease at the level of MR4.5 did not influence EFS or OS [12]. None of the patients who reached a stable MR4.5 progressed to blast phase, whereas 6 patients who did not reach a stable MR4.5 (4.4%) did progress (*p* = 0.001). We included in the analysis also drug dose, different schedules and median discontinuation time for intolerance, but all these factors did not results significant. The estimated 7-year OS was 96% in the cohort of patients with a stable MR4.5 and 89% in the cohort of patients who did not reach such a response, with a favorable trend that has so far not reached statistical significance (*p* = 0.087).

**Table 2:** Univariate analysis for the probability to achieve MR4.5

Features	Stratification	No. (%)	Probability to achieve MR4.5	*p*
Age	< 50 > 50	30% 70%	45% 33%	0.03
Gender	M F	60% 40%	31% 48%	0.02
Sokal risk	Low Interm High	48.1% 39.9% 12%	32% 22% 11%	0.01
EUTOS risk	Low High	54.8% 45.2%	47% 39%	0.067
Type of transcript	e13a2 e14a2 both	46.6% 51.9% 1.4%	31% 43% 42%	0.02
Median dose	400 mg < 400 mg	85% 15%	43% 36%	0.058
BMI	< 30 > 30	80% 20%	45% 40%	0.12
EMR at 3 months	Yes No	70% 30%	47% 21%	0.001
Median time of discontinuation for intolerance	< 15 days > 15 days	75% 25%	46% 42%	0.24
Schedule of administration	400 mg QD 200 mg BID	94% 6%	45% 43%	0.88
MMR at 12 months	Yes No	40% 60%	69% 26%	0.001

### Multivariate analysis results

We then assessed prognostic associations in a Cox multivariate regression analysis (Table 3): female gender (HR 1.6, 95% CI: 1.1–2.6; *P* = 0.022), Sokal risk (HR 1.4, 95% CI: 1.1–2.3; *p* = 0.03), type of transcript (e14a2 vs e13a2 type, HR 1.6, 95% CI: 1.3–2.9; *P* = 0.03) and achievement of EMR at 3 months (HR 1.5, 95% CI: 1.2–2.8; *P* = 0.01) were associated with a significantly higher probability of achieving MR4.5 during treatment at any time. Indeed, in multivariate analysis no association was reached for molecular responses obtained at 6 and 12 months, or for age.

**Table 3:** Multivariate regression analysis of prognostic factors associated with a stable MR4.5

Features	HR	95% CI	*P* value
Gender (female vs male)	1.6	95% CI: 1.1–2.6	0.022
Transcript type (e14a2 vs e13a2)	1.6	95% CI: 1.3–2.9	0.03
Sokal risk (low vs int/high)	1.4	95% CI: 1.1–2.3	0.03
EMR at 3 months (yes vs no)	1.5	95% CI: 1.2–2.8	0.01

### Estimation of possible candidate to discontinuation

We also estimated how many patients would satisfy criteria for discontinuation: according to Stop Imatinib trial (STIM), 29% of patients could attempt to treatment-free remission whereas according to European Stop Tyrosine Kinase Inhibitor study (EUROSKY) criteria, 42% of patients could be defined candidate [4]. Of the whole cohort, 10 patients discontinued in MR4.5 and all maintained the treatment-free remission (TFR) after a median time of 1 year.

## DISCUSSION

In this retrospective single center real life study outside of clinical trials, we could document that female gender, low vs intermediate/high Sokal risk, e14a2 type of transcript and achievement of EMR after 3 months of treatment represent prognostic factors significantly associated with a subsequent achievement of a stable MR4.5 in CML patients treated with imatinib first-line. Our results support and extend observations derived individually from clinical trials. Branford and colleagues [13] reported that in a large series of CML patients enrolled in clinical trials with imatinib the cumulative incidence of a stable MR4.5 was 36.5% after 8 years of treatment. In this study, variables associated to the achievement of a deep molecular response were female gender and an EMR obtained at 3 months (*p* < .001 for both). After 8 years 54.4% of women achieved a stable MR4.5 compared to 27.2% of men, with no differences in terms of Sokal risk, imatinib dose intensity or median age, being observed between sexes. The real reasons for this gender difference could not be defined and the phenomenon was attributed to possible pharmacokinetic differences, immune response or simply to a better adherence of females to long-term treatment [13]. We previously reported this observation [14] and now we confirm the power of this prognostic factor, which is also associated with a long-term achievement of a stable MR4.5, even when tested in a multivariate model. Moreover, we tested, according to gender, the impact of median daily dose, different schedule of administration and possible discontinuation for intolerance, but no statistical significance have been found. Indeed, a recent study measured plasma imatinib concentration and revealed a difference in mean dose normalized plasma imatinib concentrations in women as compared to men, only partially related to body weight and adherence [15]. Sokal stratification has been reported to be associated with different rates of responses in the IRIS study [2], that compared for the first time imatinib versus conventional therapy including interferon and chemotherapy: a low rate of CCyR was reported in high risk patients with a consequent reduction in EFS. The impact of Sokal risk on prognosis was also reported in the sponsored ENESTnd trial [16]: after 4 years of follow-up, only 13% of high risk patients treated with imatinib achieved MR4.5 (compared to 29% and 24% in the low and intermediate risk groups, respectively) and 32% when treated with nilotinib 300 mg BID (compared to 38% and 48% in the low and intermediate risk groups, respectively). No observations were reported regarding the possible correlation between Sokal stratification and achievement of a stable MR4.5 as documented by our analysis, confirmed also in the multivariate regression model.

The type of transcript was investigated by the MD Anderson Cancer Center group that evaluated the impact of the e14a2 and e13a2 transcripts on responses and OS [17]. Patients with the e14a2 transcript type achieved an increased and more rapid deep response with an improved EFS and treatment-free survival, but without differences in terms of OS. Also the German group reported in a large series of patients treated with imatinib that the cumulative incidence of deep molecular responses was higher in e14a2 patients [18]. An Italian cooperative group associated the transcript type to stable deep molecular response in 320 patients treated with imatinib, but a proportion of patients had been treated with interferon and the analysis was limited to MR4 and not to a complete molecular response [10].

Finally, in our regression model, the achievement of an EMR at 3 months was associated with a long-term positive outcome. This evidence confirms the value of this early evaluation as reported by several groups and with different TKIs. In particular, the Australian group showed that patients who had achieved at 3 months a BCR-ABL1 ratio < 0.1% had a significantly higher cumulative incidence of stable MR4.5 after 8 years (78.2%) compared to patients with a ratio > 0.1% and < 1% (52.7%) or to patients who had a ratio > 1% up to 10% (29%). Patients who had not achieved an EMR at 3 months had the lowest percentage of stable MR4.5 after 8 years (8.6%) [13]. The same value was reported also in patients treated upfront with second generation TKIs: in the ENESTnd trial, considering MR4.5 at 4 years of follow-up, this was achieved only in 4% of patients treated with nilotinib without EMR at 3 months and in 5% of patients treated with imatinib with a similar degree of response [16]. Also with dasatinib a ratio < 10% at 3 months was reported to be associated with an increased rate of MR4.5 at 2 years [19]. At the last follow-up of the DASISION trial, the rate of patients who achieved a MR4.5 according to the ratio at 3 months was reported: of patients who achieved an EMR at 3 months, 54% reached a deep response compared to only 5% of patients who did not achieve an early response EMR at 3 months [20]. Also in our real life study with imatinib first-line the same effect was observed: 48% of patients who obtained an EMR at 3 months achieved a MR4.5 at 5 years, while only 12% of patients who did not reach an EMR obtained a MR4.5.

A stable deep molecular response has become an endpoint for the majority of CML patients in order to attempt a possible treatment discontinuation. A long follow-up of the STIM1 study has shown that only about 40% of patients remained disease-free without imatinib after 65 months [21]. The early identification of possible candidates to a treatment-free remission is now a primary challenge in the management of CML. The results of our study conducted in a real life setting indicate that well defined clinical and biologic factors can help to better identify those patients who are more likely to benefit from the treatment discontinuation option.

## MATERIALS AND METHODS

### Patient population

A series of 208 chronic phase CML patients treated first-line with imatinib at our center outside of clinical trials and having a median follow-up of 7 years was analyzed. Patients were diagnosed and followed between January 2000 and December 2014. No patient had received interferon prior to imatinib treatment.

### Response categories and monitoring

The following response categories were analyzed using standard definitions: CCyR, major molecular response (MR3 or ratio BCR-ABL/ABL < 0.1% IS), MR4 (ratio BCR-ABL/ABL < 0.01% IS) and MR4.5 (ratio BCR-ABL/ABL < 0.0032% IS). Patients were monitored and stratified at each time point according to the 2013 European LeukemiaNet (ELN) recommendations [11]. A stable deep molecular response has been defined as a confirmation of MR4.5 in two consecutive samples. Fisher’s exact test and Chi-square with Yate’s correction were performed.

### Statistical analysis

The following features were included in the analysis aimed at identifying the probability of obtaining a stable deep molecular response: age, gender, Sokal and Eutos score, white blood cell count, hemoglobin level, platelet count, BCR-ABL transcript type, type of response at each time point according to the 2013 ELN recommendations [11]. First, a univariate analysis was performed to evaluate the predictive effect of each factor alone, trough SPSS tool version 10.0. Then, any factor with a *p* value < 0.05 that emerged from the univariate test was selected to construct a full multivariate Cox regression model to predict baseline prognostic factors for the entire study cohort. The full model was reduced to a final model using the stepwise selection method so that all factors remaining in the model were statistically significant. EFS was measured from the start of treatment to the occurrence of an event, considering loss of complete hematologic response (CHR) or major cytogenetic response, progression to advanced phase of disease (accelerated or blast phase), or death from any cause during treatment. OS was defined from the date of CML diagnosis to the date of death or last follow-up using the Kaplan Meyer method.
